# Basiliximab vs. No Induction Therapy in Kidney Transplant Recipients with a Low Immunological Risk Profile Receiving Tacrolimus/Mycophenolate/Steroids Maintenance Immunosuppression

**DOI:** 10.3390/jcm13206151

**Published:** 2024-10-16

**Authors:** Florian Lacave, Christophe de Terwangne, Tom Darius, Antoine Buemi, Michel Mourad, Yannick France, Joana Cardoso Coelho, Guillaume Fernandes, Eric Goffin, Arnaud Devresse, Nada Kanaan

**Affiliations:** 1 Division of Nephrology, Cliniques Universitaires Saint-Luc, Université Catholique de Louvain, Avenue Hippocrate 10, 1200 Brussels, Belgium; florian.lacave@saintluc.uclouvain.be (F.L.); guillaume.fernandes@saintluc.uclouvain.be (G.F.); eric.goffin@uclouvain.be (E.G.);; 2 Geriatrics Medecine, Cliniques Universitaires Saint-Luc, Université Catholique de Louvain, Avenue Hippocrate 10, 1200 Brussels, Belgium; christophe.deterwangne@saintluc.uclouvain.be; 3 Institut de Recherche Experimentale et Clinique (IREC), Cliniques Universitaires Saint-Luc, Université Catholique de Louvain, Avenue Hippocrate 10, 1200 Brussels, Belgium; 4 Surgery and Abdominal Transplant Unit, Cliniques Universitaires Saint-Luc, Université Catholique de Louvain, Avenue Hippocrate 10, 1200 Brussels, Belgium; tom.darius@saintluc.uclouvain.be (T.D.); antoine.buemi@saintluc.uclouvain.be (A.B.); michel.mourad@uclouvain.be (M.M.); yannick.france@saintluc.uclouvain.be (Y.F.); joana.cardoso@saintluc.uclouvain.be (J.C.C.)

**Keywords:** induction, kidney transplantation, acute rejection, graft loss

## Abstract

**Background**: Induction therapy with basiliximab is recommended in kidney transplant (KT) recipients with a low immunological risk (LIR) profile. Whether basiliximab is associated with a decreased risk of acute rejection (AR) and graft loss is controversial. **Methods**: In our institution, LIR patients (absence of anti-HLA antibodies before KT) are inducted with basiliximab in case of living-donor KT, while deceased-donor KT recipients receive no induction. Maintenance immunosuppression is similar, including a combination of tacrolimus (Tac), mycophenolate (MPA) and steroids. In this single-center retrospective study, we included all adult LIR patients who underwent KT between 1 January 2015 and 31 December 2022. **Results**: Of the 471 patients included, 354 received no induction and 117 received basiliximab. The median (IQR) number of HLA A-B-DR mismatches was 3 (2–3) and 2 (2–4) in the no induction group and the basiliximab group, respectively. The cumulative incidences in the no induction group vs. the basiliximab group of acute rejection and graft loss over 5 years post-KT were similar at 8.9% vs. 7.8% (*p* = 0.8) and 8.5% vs. 4.2% (*p* = 0.063), respectively. In multivariable Cox regression analysis, delayed graft function emerged as an independent risk factor for acute rejection (hazard ratio [HR] 2.75, 95% confidence interval [CI] 1.23–6.13, *p* = 0.014) and graft loss (HR 9.32, CI 4.10–21.1, *p* < 0.001). **Conclusions:** Basiliximab did not provide any advantage in terms of rate of acute rejection and graft survival within 5 years post KT compared with a strategy without induction therapy in patients with a low immunological risk profile receiving triple maintenance immunosuppression Tac/MPA/steroids.

## 1. Introduction

Induction therapy with antilymphocyte biological agents (mostly T lymphocyte-depleting rabbit-derived antithymocyte globulin [rATG] and an IL-2 receptor antagonist [IL2RA]) is widely used as part of the initial immunosuppressive treatment in kidney transplantation (KT).

However, the benefit of induction therapy in patients with a low immunological risk (LIR) profile is a matter of debate [1]. The use of induction therapy in early trials has been associated with a lower likelihood of acute rejection, but this occurred within the framework of outdated immunosuppressive protocols [2,3]. Indeed, no randomized controlled trial has assessed the effectiveness of a systematic induction therapy in LIR patients receiving the standard immunosuppressive regimen combining tacrolimus (Tac), mycophenolate (MPA) and steroids. Several retrospective and registry studies have demonstrated little to no advantage of induction therapy compared to no induction in low-immunological-risk patients who are administered a combination of Tac, MPA, and steroids with regard to the survival of the transplanted organ [4,5,6,7,8].

In this study, we aimed at comparing the 1- and 5-year risk of acute graft rejection, graft survival and patient survival between IL2RA induction versus no induction in a cohort of kidney transplant recipients with a low immunological risk profile receiving Tac/MPA/steroid maintenance immunosuppressive therapy.

## 2. Methods

### 2.1. Patient Selection

All adults (>18 years-old) with a LIR profile, transplanted between 1 January 2015 and 31 December 2022, were included. Patients with a historic virtual panel reactive antibody [vPRA 0%]) were considered as LIR profile. Patients with a history of vPRA > 0%, those who underwent an ABO incompatible KT or multiorgan transplantation and those who received specific peritransplant management (e.g., plasma exchanges to prevent focal segmental glomerulosclerosis recurrence or prophylactic eculizumab in moderate and high-risk atypical hemolytic and uremic syndrome) were excluded.

The study adhered to the principles of the Declaration of Helsinki and was approved by the ethics committee of the Cliniques Universitaires Saint-Luc (Reference 2022/22JUL/288). The clinical and research activities being reported are consistent with the Principles of the Declaration of Istanbul as outlined in the ‘*Declaration of Istanbul on Organ Trafficking and Transplant Tourism*’.

### 2.2. Management of Immunosuppression and Surveillance after Transplantation

We apply a standard protocol regarding induction therapy in LIR patients. Deceased-donor KT recipients (DDKTs) receive no induction, while basiliximab is given (20 mg at day 0 and day 4) in patients who receive a living-donor KT. Maintenance immunosuppression is similar for all patients, including a combination of Tac (a trough level range of 9–13 ng/mL during the first month after KT, 7–9 ng/mL in months 2 and 3, and 5–7 ng/mL thereafter), MPA (500 mg bid) and steroids (methylprednisolone 500 mg at day 0, 16 mg/day from D1 with reduction of 4 mg every two weeks to reach a daily dose of 4 mg/day, continued lifelong). All KT recipients receive prophylactic treatment including co-trimoxazole (3 times/week) and valganciclovir (except in *Cytomegalovirus* [CMV] serostatus D-/R-) for 6 months.

Human leucocytes antigen (HLA) antibody and BK virus infection are screened every 3 months during the first year post KT, and then annually or in case of clinical suspicion (e.g., acute allograft dysfunction). Kidney biopsy is performed in case of acute allograft dysfunction, new onset proteinuria, hematuria, or de novo donor-specific antibody (dnDSA) emergence.

### 2.3. Data Collection

The following baseline data were collected: demographics (gender, age), pre-transplant history of diabetes, cardiovascular events (stroke, coronary or peripheral arterial disease), solid-organ transplant or neoplasia (excluding local basal cell carcinoma), dialysis vintage, cause of kidney failure, CMV status and transplantation data (date of transplantation, rank of transplantation, donor source, vPRA, HLA A-, B-, DR- number of mismatches between the donor and the recipient, ischemia time, induction therapy, maintenance immunosuppressive treatment).

After transplantation, the following data were collected: delayed graft function (DGF) (defined as the need for at least one hemodialysis session—except for hyperkalemia—in the first week post transplant), biopsy-proven acute rejection (BPAR) episode (histology according to the 2022 Banff classification treatment [9]), de novo donor specific antibody (dnDSA) emergence, serum creatinine and estimated glomerular filtration rate (eGFR) (calculated with CKD EPI formula) at different time points (1, 3 and 12 months after KT and at last follow-up), graft loss and related cause (defined as a return to dialysis or retransplantation) and death and related cause. In this study, HLA antibody assessments performed at the time of an acute rejection episode, at 1 and 2 years post transplant and every 2 years thereafter, were recorded.

The data were collected until graft loss, patient death or data cut-off (31 March 2024).

### 2.4. Statistics

Statistical analysis was performed using R (version 4.3.2), R Core Team, Vienna, Austria, 2022. Categorical variables were expressed as counts with percentages and continuous variables as mean (±SD) or median [25th–75th percentiles] as appropriate. Differences in patient characteristics between those without induction therapy (deceased donors) and patients receiving basiliximab (living donors) were assessed using the χ^2^ test or Fischer Exact test for categorical variables and Student’s *t*-test or the Wilcoxon rank-sum test for continuous variables as appropriate. Estimations of probabilities of outcome (acute rejection, graft loss, or infection) over time are modeled using cumulative incidence curves (1-Kaplan–Meier estimate) and differences in the probability of occurrence of an outcome between treatment groups were computed using the log-rank test. To adjust the effect of each treatment group (no induction and basiliximab) on different outcomes (acute rejection, graft loss or infection), multivariable Cox proportional hazard regression models were computed. Each model was adjusted for demographic variables (age and gender), on one hand, and variables with *p*-values of <0.15 in univariable analysis, on the other hand. Collinear variables, i.e., with evident clinical correlation or a variance inflation factor (VIF) greater than five, were excluded before multivariable modeling.

## 3. Results

### 3.1. Baseline Characteristics

**Figure 1:**
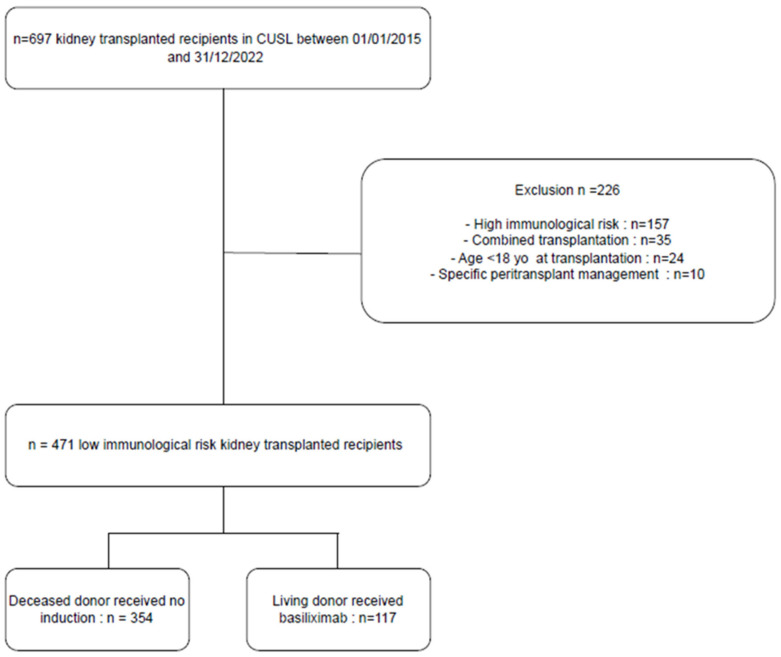
Flowchart of the study.

Of the 697 patients who received a kidney transplant in our center during the study period, 471 patients with LIR were included (Figure 1). Among them, 354 (75%) were transplanted from a deceased donor with no induction therapy (“no induction group”) and 117 (25%) were transplanted from a living donor with basiliximab as induction therapy (“basiliximab group”).

At transplantation, median age was 53 years and 341 (72%) patients were male. Compared to patients in the basiliximab group, those in the no induction group were more frequently male (76% vs. 62%), older (56 [interquartile range (IQR) 46–64] years vs. 48 [IQR 34–55]) years, had more frequently a history of cardiovascular event and diabetes (29% vs. 13% and 26% vs. 14%, respectively), received fewer preemptive KT (5.9% vs. 35%), had a longer pre-KT time spent on dialysis (40 months [IQR 23–60] vs. 15 months [IQR 8–23]), and a longer cold ischemia time (10 [IQR 7–14] h vs. 3 [IQR 2–3] h). The median number of HLA A-B-DR mismatches was similar in the no induction group and the basiliximab group (3 [IQR 2–3] and 2 [IQR 2–4], *p* = 0.6, respectively). In the no induction group, 70% received a graft from a donor after brain death (DBD) while 30% received a graft from a donor after circulatory death (DCD) (Table 1).

The median follow-up time was 4.05 [2.2–6.1] years for the no induction group and 4.92 [2.07–7.05] years for the basiliximab group.

**Figure 2:**
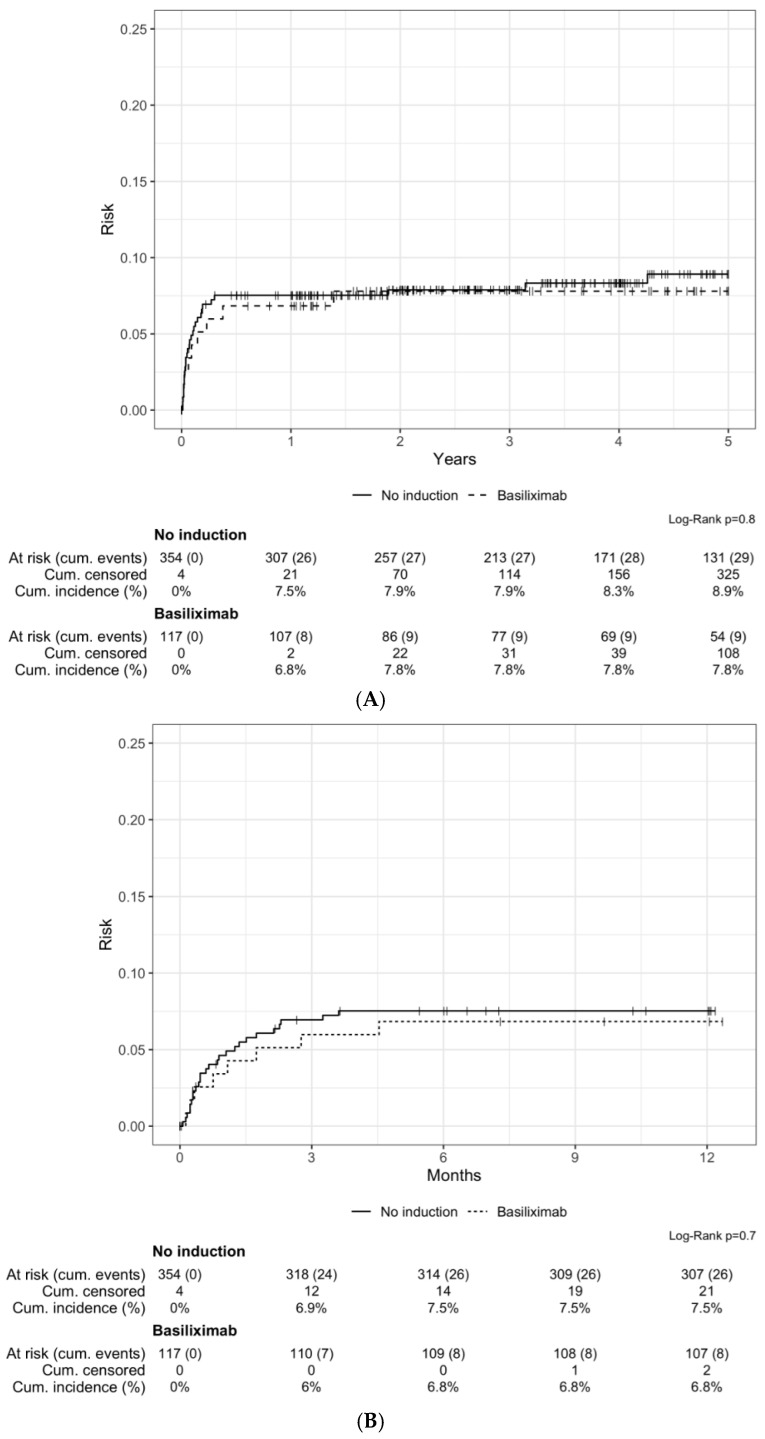
Cumulative incidence of acute rejection over 5 years (**A**) and over 1 year (**B**) after transplantation.

Biopsy-proven acute rejection (BPAR) occurred in 38 patients (8.6%) during the first five years post KT. The cumulative incidence of BPAR at one year was similar in both groups (7.5% [n = 26] in the no induction group vs. 6.8% [n = 8] in the basiliximab group, *p* = 0.7) as was the cumulative incidence over 5 years (8.9% [n = 29] in the no induction group vs. 7.8% [n = 9] in the basiliximab group, *p* = 0.8) (Figure 2A). The majority of BPARs occurred early after transplantation, in the first trimester, in both groups (Figure 2B). The most frequent types of rejection were T-cell-mediated rejection (TCMR) and borderline rejection in the no induction group (45% [n = 13] and 38% [n = 11], respectively), while in the basiliximab group, borderline rejection (44% [n = 4]) prevailed. Three patients with TCMR Banff grade I rejection were treated with corticosteroids; 12 patients (both groups included) with more severe TCMR were treated with rATG and corticosteroids. Borderline rejections were treated with corticosteroids. We observed two cases of antibody-mediated rejections (AMRs) in the no induction group and 1 in the basiliximab group, all treated with plasma exchange and steroids (and rituximab for the patient from the basiliximab group). The incidence of de novo DSA in the first 5 years post transplant was low and similar in both groups (three [0.8%] in the no induction group and two [1.7%] in the basiliximab group).

Table 2 shows a Cox regression aiming at assessing independent risk factors for acute rejection occurrence. Multivariable analysis revealed that delayed graft function (DGF) was an independent risk factor of BPAR (HR 2.75, 95% confidence interval [CI] 1.23–6.13, *p* = 0.014), while recipient age was protective (HR 0.98, CI 0.95–1.00, *p* = 0.044).

### 3.2. Graft Function and Graft Survivals

**Figure 3:**
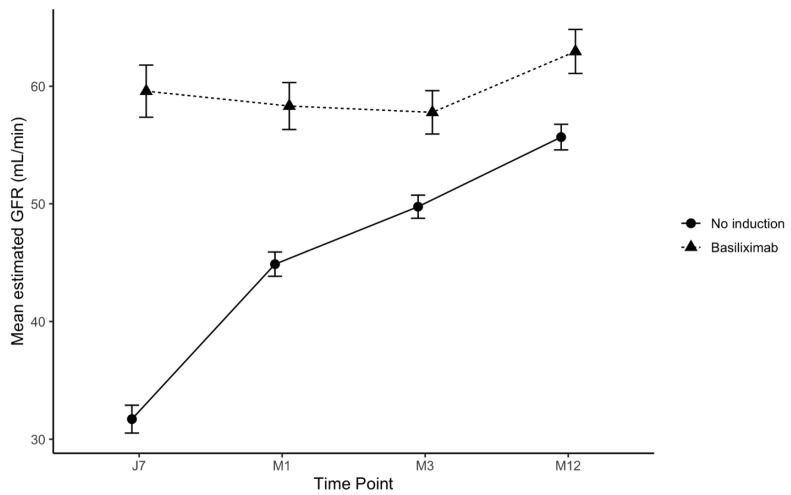
Trends in eGFR in the first year after transplantation.

Different trends in eGFR post transplantation have been observed between the two groups (Figure 3). Mean eGFR at one week after the transplantation was 31.7 ± 1.2 mL/min/1.73 m^2^ in the no induction group vs. 59.6 ± 2.2 mL/min/1.73 m^2^ in the basiliximab group. At 12 months after KT, mean eGFR increased to 55.7 (±1.1) mL/min/1.73 m^2^ in the no induction group and to 63.0 (±1.9) mL/min/1.73 m^2^ in the basiliximab group.

**Figure 4:**
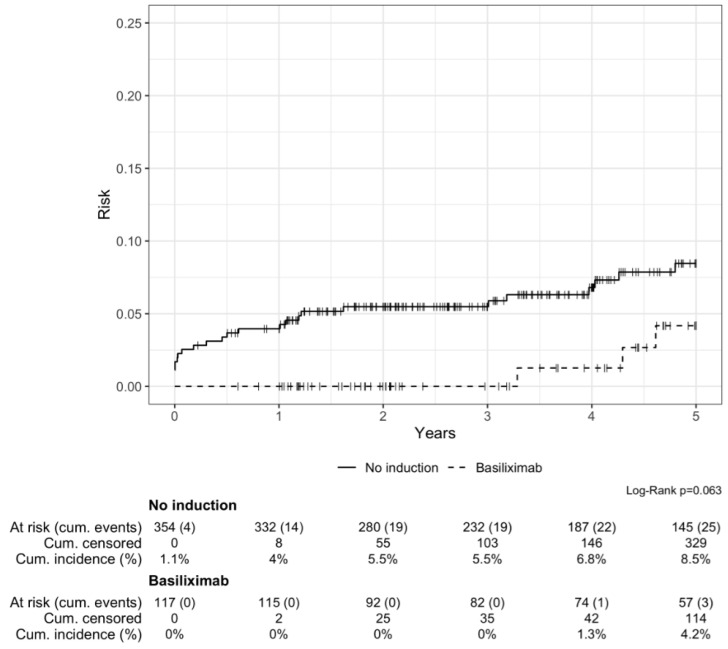
Cumulative incidence of graft loss over 5 years after transplantation.

Graft loss occurred in 28 patients (7.4%) within the first 5 years post KT. The cumulative incidence of graft loss over 5 years was not significantly different in both groups (8.5% in the no induction group vs. 4.2% in the basiliximab group, *p* = 0.063) (Figure 4).

In univariable analysis, a protective trend was observed in case of basiliximab (HR 0.34 *p* = 0.076), but significance was lost after adjustment (HR 0.82, *p* = 0.8) (Table 3). However, DGF and a history of pre-transplant neoplasia emerged as independent risk factors in the multivariable Cox regression model for graft loss (HR 9.32, CI 4.10–21.1, *p* < 0.001 and HR 3.28, CI 1.29–8.32, *p* = 0.012; respectively).

## 4. Discussion

The results of our study show that, compared with no induction, basiliximab is not associated with a significant clinical benefit in terms of acute rejection occurring during the first year post transplant and at 5-year follow-up, development of de novo DSA and graft or patient survival, in KT recipients with a low immunological risk profile receiving a combination of Tac/MPA/steroids maintenance immunosuppression. Our results also reveal that delayed graft function is a strong independent risk factor for acute rejection and graft loss.

Several registry analyses have previously shown the absence of the significant impact of basiliximab in LIR KT recipients. Using the Organ Procurement and Transplantation Network/Scientific Registry of Transplant Recipients (OPTN/STR) registry data, Tanriover et al. found, in a large cohort of living-donor KT recipients (n = 36.153, inclusion criteria: maximum one prior KT, no identical HLA, only uniorgan transplantation), that basliximab induction showed similar outcomes regarding the risk of acute rejection at 1 year and overall allograft failure, than no induction in patients treated by Tac, MPA and steroids [4]. Also, with the use of the OPTN/UNOS database, Sureshkumar et al. compared the outcomes of groups of low-immunological-risk KT recipients (defined as first transplant, panel reactive antibody < 20%, human leukocyte antigen mismatches ≤ 3) [5]. Each group contained recipients of mate kidneys (from the same deceased donor) and differed by the induction received: basliximab vs. no induction, no induction vs. depleting antibody induction, and depleting antibody induction vs. basiliximab. Outcomes were relatively comparable between mate-kidney recipients in each group. Notably, adjusted five-year graft survivals were similar between mate-kidney recipients in all three groups. More recently, Evans et al., again with the use of the OPTN registry, reported the outcomes of first adult kidney-only transplant recipients in the United States, who were well matched with their donor at HLA-A, -B, -DR, -DQB1 on the basis of serologic typing [6]. The study included 2976 patients inducted by T-cell-depleting antibodies (57%), basiliximab (28%) or no induction (15%). They found that induction therapy was not associated with improved outcomes in terms of graft survival, death-censored graft survival or death with function. Our study is in line with these reports and other retrospective studies [7,8]. Indeed, in our cohort, the cumulative incidence of acute rejection at 5 years was very similar between the no induction and the basiliximab group (8.9% vs. 7.8%). As expected, most BPARs occurred in the first trimester, with a low rate of 6% in both groups, reflecting the efficacy of the current standard immunosuppressive triple therapy comprising tacrolimus/MPA/steroids. The cumulative incidence of death-censored graft loss at 5 years was higher in the no induction group, but was not statistically significant (8.5% vs. 4.2%; *p* = 0.063). As acute rejection rates were similar in both groups, this difference may be explained by the donor characteristics—living donors in the basiliximab group vs. deceased donors in the no induction group—resulting in better eGFR at 5 years (62 mL/min, vs. 55 mL/min) [10].

Analyzing independent risk factors for acute rejection, we found recipient age to be protective. This is in agreement with the reported literature that attributes the reduced incidence of acute rejection in older individuals to immunosenescence and underscores treatment non-adherence as associated with cellular and antibody-mediated rejection in younger recipients [11,12]. Also, in agreement with the literature, we found DGF to be independently associated with acute rejection and graft loss. Wu et al. have shown in a cohort study of 645 patients that DGF was associated with a 52% and 54% increased risk of developing T-cell rejection and antibody-mediated rejection, respectively [13]. In addition, a recent meta-analysis revealed that patients who experienced DGF had >3-fold higher risk of developing graft failure compared with those who did not [14].

Induction therapy is still largely used worldwide in low-immunological-risk patients. Indeed, the OPTN/SRTR data report recently revealed that approximately 92% of adult KT recipients in 2022 received induction therapy in the US, regardless of their immunological risk [10]. This is in agreement with the last *Kidney Disease: Improving Global Outcomes* (KDIGO) guidelines for kidney transplantation, published in 2009, that strongly recommend its use [15]. Consequently, there is a need to refresh international guidelines and assess the safety and efficacy of basiliximab in low-immunological-risk patients in future large prospective and randomized trials. Indeed, in addition to cost issues, it is not excluded that basiliximab might be associated with clinical consequences. It is well known that compared to depleting antibody induction, basiliximab is associated with a lower risk of post-transplant malignancy [16,17,18,19] and infection [16,20,21]. However, the evidence of the safety of basiliximab is scarcer when compared with no induction, because of the paucity of data. This important question should also be assessed in future clinical trials.

Our study’s strengths lie in the relatively large cohort size and the comprehensive, well-conducted follow-up of all patients, which differentiates it from registry studies that lack granularity. We acknowledge however its limitations. Its retrospective nature makes it subject to unexpected bias. Also, we defined the low-immunological-risk profile as patients with a historic vPRA 0%. No robust definition of the LIR status exists and a large heterogeneity of definitions are used in clinical studies. KDIGO guidelines only describe risk factors for acute rejection after kidney transplantation such as HLA mismatch, PRA > 0%, older donor age or younger recipient age [15]. This lack of consensus makes interpretation of data complex and international societies should define a single definition for use in future clinical trials. Finally, because of the nature of our local protocol regarding induction therapy, it is not excluded that donor characteristics have influenced our results. Indeed, only living-donor KT patients received basiliximab, while only deceased-donor KT patients were not inducted. This accounts most likely for the better (but not significant) 5-year graft survival. Indeed, it is well known that living-donor KT is associated with a graft survival advantage compared to deceased-donor KT [22]. However, it is unlikely that this donor discrepancy had an impact on the risk of acute rejection or DSA development in our cohort, which was well balanced regarding HLA mismatch—known to be a strong risk factor for the occurrence of acute rejection [23].

In conclusion, our study shows that induction therapy with basiliximab has no benefit regarding the risk of acute rejection at one year and mid-term follow-up, or graft loss within 5 years post KT compared to a strategy without induction therapy in patients with a low immunological risk profile who receive Tac/MPA/steroids maintenance therapy. Further large prospective studies are needed to confirm our results and provide strong evidence to allow a change in the recommendations elaborated by international guidelines.

## Figures and Tables

**Table 1:** Baseline characteristics.

	Overall, N = 471 ^1^	No Induction, N = 354 ^1^	Basiliximab, N = 117 ^1^	*p*-Value ^2^
Gender				0.005
Man	341 (72%)	268 (76%)	73 (62%)	
Age (years)	53 (43, 63)	56 (46, 64)	48 (34, 55)	<0.001
Kidney disease				0.2
Congenital or hereditary nephropathy	139 (30%)	102 (29%)	37 (32%)	
Diabetes	67 (14%)	56 (16%)	11 (9.4%)	
Glomerulopathy	102 (22%)	68 (19%)	34 (29%)	
Interstitial nephropathy	43 (9.1%)	33 (9.3%)	10 (8.5%)	
Other/Unknown	100 (21%)	79 (22%)	21 (18%)	
Vascular nephropathy	20 (4.2%)	16 (4.5%)	4 (3.4%)	
Vintage dialysis (months)	35 (19, 56)	40 (23, 60)	15 (8, 23)	<0.001
Dialysis mode				<0.001
Hemodialysis	374 (79%)	307 (87%)	67 (57%)	
Peritoneal dialysis	35 (7.4%)	26 (7.3%)	9 (7.7%)	
Preemptive	62 (13%)	21 (5.9%)	41 (35%)	
Prior cardiovascular events	116 (25%)	101 (29%)	15 (13%)	<0.001
Prior diabetes	107 (23%)	91 (26%)	16 (14%)	0.007
Prior neoplasia	53 (11%)	45 (13%)	8 (6.8%)	0.081
Prior organ transplantation (non-kidney)	16 (3.4%)	10 (2.8%)	6 (5.1%)	0.2
Number of kidney transplantation				0.10
1	449 (95%)	339 (96%)	110 (94%)	
2	20 (4.2%)	15 (4.2%)	5 (4.3%)	
3	2 (0.4%)	0 (0%)	2 (1.7%)	
Maintenance therapy				0.6
Tacrolimus/mycophenolate mofetil/steroids	468 (99%)	352 (99%)	116 (99%)	
Number of HLA mismatch	3.00 (2.00, 3.00)	3.00 (2.00, 3.00)	2.00 (2.00, 4.00)	0.6
Cold ischemia (hours)	9.0 (4.0, 13.0)	10.0 (7.0, 14.0)	3.0 (2.0, 3.0)	<0.001
CMV status				0.001
Low-risk	109 (23%)	95 (27%)	14 (12%)	
Intermediate risk	260 (55%)	180 (51%)	80 (68%)	
High-risk	102 (22%)	79 (22%)	23 (20%)	

Acute rejection and de novo DSA. ^1^ n (%), median (IQR) ^2^ Pearson’s chi-squared test; Wilcoxon rank-sum test; Fisher’s exact test.

**Table 2:** Univariable and multivariable Cox regression for acute rejection.

	Unadjusted			Adjusted		
	HR ^1^	95% CI ^1^	*p*-Value	HR ^1^	95% CI ^1^	*p*-Value
**Induction**						
No induction	—	—		—	—	
Basiliximab	0.90	0.43, 1.90	0.8	0.62	0.25, 1.53	0.3
**Gender**						
Man	—	—		—	—	
Woman	1.41	0.72, 2.76	0.3	1.38	0.69, 2.76	0.4
**Age**	0.98	0.96, 1.00	**0.049**	0.98	0.95, 1.00	**0.044**
**Prior dialysis**						
Dialysis	—	—		—	—	
Preemptive	1.74	0.80, 3.79	0.2	2.30	0.93, 5.65	0.070
**Number of KT**	1.89	0.77, 4.67	0.2			
**Prior CV event**	0.71	0.31, 1.60	0.4			
**Prior diabetes**	0.52	0.20, 1.33	0.2			
**Prior neoplasia**	1.28	0.50, 3.29	0.6			
**Prior transplantation**	0.77	0.11, 5.58	0.8			
**Cold ischemia**	1.01	0.95, 1.07	0.8			
**CMV status**						
Low-risk	—	—				
Intermediate risk	1.82	0.75, 4.43	0.2			
High-risk	1.05	0.34, 3.24	>0.9			
**DGF**	2.36	1.11, 4.98	**0.025**	2.75	1.23, 6.13	**0.014**

^1^ HR = hazard ratio, ^1^ CI = confidence interval, bold = *p* < 0.05.

**Table 3:** Univariable and multivariable Cox regression for graft loss.

	Unadjusted			Adjusted		
	HR ^1^	95% CI ^1^	*p*-Value	HR ^1^	95% CI ^1^	*p*-Value
**Induction**						
No induction	—	—		—	—	
Basiliximab	0.34	0.10, 1.12	0.076	0.82	0.23, 2.99	0.8
**Gender**						
Man	—	—				
Woman	1.06	0.47, 2.41	0.9			
**Age**	1.02	0.99, 1.05	0.3			
**Prior dialysis**						
Dialysis	—	—				
Preemptive	0.44	0.10, 1.85	0.3			
**Number of KT**	0.71	0.11, 4.70	0.7			
**Prior CV event**	2.56	1.21, 5.43	**0.014**			
**Prior diabetes**	2.16	0.99, 4.69	0.052	1.69	0.76, 3.76	0.2
**Prior neoplasia**	2.38	0.97, 5.88	0.060	3.28	1.29, 8.32	**0.012**
**Prior transplantation**	1.07	0.15, 7.86	>0.9			
**Cold ischemia**	1.06	0.99, 1.13	0.074			
**CMV status**						
Low-risk	—	—				
Intermediate risk	1.81	0.61, 5.35	0.3			
High-risk	1.54	0.43, 5.46	0.5			
**DGF**	9.37	4.44, 19.8	**<0.001**	9.32	4.10, 21.1	**<0.001**

^1^ HR = hazard ratio, ^1^ CI = confidence interval, bold = *p* < 0.05.

## Data Availability

The raw data supporting the conclusions of this article will be made available by the authors on request.
